# Osteogenic Differentiation of Human Mesenchymal Stem Cells in 3-D Zr-Si Organic-Inorganic Scaffolds Produced by Two-Photon Polymerization Technique

**DOI:** 10.1371/journal.pone.0118164

**Published:** 2015-02-23

**Authors:** Anastasia Koroleva, Andrea Deiwick, Alexander Nguyen, Sabrina Schlie-Wolter, Roger Narayan, Peter Timashev, Vladimir Popov, Viktor Bagratashvili, Boris Chichkov

**Affiliations:** 1 Nanotechnology Department of Laser Zentrum Hannover, Hannover, Germany; 2 Joint Department of Biomedical Engineering, North Carolina State University, Raleigh, North Carolina, United States of America; 3 Institute of Laser and Information Technologies, Moscow, Russia; Universita’ degli Studi del Salento, ITALY

## Abstract

Two-photon polymerization (2PP) is applied for the fabrication of 3-D Zr-Si scaffolds for bone tissue engineering. Zr-Si scaffolds with 150, 200, and 250 μm pore sizes are seeded with human bone marrow stem cells (hBMSCs) and human adipose tissue derived stem cells (hASCs) and cultured in osteoinductive and control media for three weeks. Osteogenic differentiation of hASCs and hBMSCs and formation of bone matrix is comparatively analyzed via alkaline phosphatase activity (ALP), calcium quantification, osteocalcin staining and scanning electron microscopy (SEM). It is observed that the 150 μm pore size Zr-Si scaffolds support the strongest matrix mineralization, as confirmed by calcium deposition. Analysis of ALP activity, osteocalcin staining and SEM observations of matrix mineralization reveal that mesenchymal stem cells cultured on 3-D scaffolds without osteogenic stimulation spontaneously differentiate towards osteogenic lineage. Nanoindentation measurements show that aging of the 2PP-produced Zr-Si scaffolds in aqueous or alcohol media results in an increase in the scaffold Young’s modulus and hardness. Moreover, accelerated formation of bone matrix by hASCs is noted, when cultured on the scaffolds with lower Young’s moduli and hardness values (non aged scaffolds) compared to the cells cultured on scaffolds with higher Young’s modulus and hardness values (aged scaffolds). Presented results support the potential application of Zr-Si scaffolds for autologous bone tissue engineering.

## Introduction

Tissue engineering (TE) scaffolds must provide a cell environment similar to that of native tissues. They should be produced by a method that enables control over the scaffold architecture and chemical composition in order to influence cellular behavior in a desired manner. Depending on the tissue engineering application scaffold structures must exhibit certain geometrical properties in order to permit 3-D cell infiltration and growth. Therefore, precise methods for the fabrication of TE scaffolds with well-defined pore sizes and predictable interconnectivities are highly desired [1]. With the rapid advancement of nanotechnology and particularly contact-free computer-aided microfabrication methods, it has become possible to produce biomimetic synthetic scaffolds with high precision and reproducibility [2,3]. These abilities can be found in laser-based microfabrication technologies which are known for high resolution spatial control of the generated structures. Two-photon polymerization (2PP) fulfills the above requirements and enables the fabrication of 3-D microstructures with complex architectures and precise dimensions [4–6]. This process uses simultaneous absorption of two photons of near-infrared (780 nm) or green (515 nm) laser light, which takes place at high laser intensity within a spatially localized focus region. If a NIR transparent and UV sensitive photopolymer is used, absorption and polymerization occur only at the focal spot. The 2PP-fabricated microstructures are characterized by high fidelity with the corresponding computer-generated designs. 2PP allows reproducible fabrication of more precise matrices mimicking cellular biological environments.

In this paper, 2PP fabrication of porous 3-D structures from Zr-Si-based organic-inorganic hybrid materials and their use as scaffolds for stem cell based orthopedic applications is described. The scaffolds with 150, 200 and 250 μm pore sizes were fabricated by 2PP and have been investigated in terms of their mechanical properties, stem cell seeding efficiency, cell proliferation, and induction of differentiation towards osteogenic lineage.

Ceramic materials have been used for decades in dental restoration and their performance for this application remains irreplaceable. Bioactive ceramics have been introduced as coatings on implant surface to enhance the ingrowth of bone tissue into the implant [7]. Taking into account earlier successful applications of ceramic materials in reconstructive therapy, porous 3-D polymer ceramic scaffolds can be good candidates for the engineering of hard tissues (e.g., teeth and bone).

Photosensitive Zr-Si-based inorganic-organic hybrid materials were synthesized via sol gel process [8]. This organically modified ceramics, which exhibits high Zr content, was previously utilized for 2PP fabrication of photonic microstructures [9–11]. Zr-Si ceramic ‘cell-culture friendly’ environment has been established by Psycharakis et al. using 3T3 mouse fibroblasts. It was suggested that the organic part of the material provides the soft matrix for cell growth, whereas the inorganic component provides the mechanical stability and rigidity of the 3-D structures [12]. Using MC3T3–E1 pre-osteoblast cell line, Terzaki et al. have explored the application of Zr-Si-based inorganic-organic hybrid materials for bone tissue engineering. They found that cell proliferation and viability on flat Zr-Si films were comparable with controls and that combination of Zr-Si 3-D surface structures with aspartate-containing self-assembling peptides targeted for calcium binding improves the bone specific ECM mineralization [13,14]. Raimondi et al. have used Zr-Si-based inorganic-organic hybrid materials to fabricate arrays of different niche architectures for culturing rat mesenchymal stem cells. It was observed that the highest cell proliferation occurred in the regions of highest scaffold surface density available for cell adhesion [15]. Recently, our group has reported on culturing of human bone marrow stem cells on surface grid structures fabricated by 2PP from Zr-Si-based inorganic-organic hybrid materials. It was shown that the cell orientation is dependent on the pattern microarchitecture. This study demonstrated the potential of zirconium oxide hybrid material-based 3-D microarchitectures for long-term growth of hBMSCs [16].

Despite the above mentioned studies, the biological performance of Zr-Si-based composites with respect to osteogenic differentiation of human mesenchymal stem cells has not been well examined. The potential of Zr-Si scaffolds to trigger stem cells specifically to osteogenic differentiation and identification of optimal pore sizes for improved osteogenesis were the goals of this study. The osteogenic differentiation potential of human bone marrow stem cells (hBMSCs) and human adipose-derived stem cells (hASCs) seeded on the Zr-Si scaffolds was comparatively studied. Both stem cell types are known for their self-renewal capacity and multi-lineage differentiation potential, representing an attractive cell source for tissue regeneration. It has been reported that hBMSCs and hASCs are not a homogeneous population of multilineage progenitor cells, but rather a heterogeneous population of pluripotent stem cells as well as tripotent, bipotent, and unipotent progenitor cells [17,18]. To further develop Zr-Si scaffolds for bone tissue engineering, a stem cell source with the highest osteogenic potential and optimal scaffold pore sizes should be identified. This knowledge could facilitate the development of improved scaffolds for autologous bone tissue engineering.

## Materials and Methods

### Synthesis of Zr-Si-based organic inorganic polymer ceramic composites

Zr-Si inorganic-organic hybrids were prepared according to a procedure that was previously described [8,11]. The sol-gel reaction was performed in three steps. First, the inorganic precursor 3-methacryloxypropyltrimethoxysilane (MAPTMS, Assay 99% in methanol, Sigma-Aldrich) was hydrolyzed using a 0.01 M HCl solution. Simultaneously, in a separate container, zirconium (IV) n-propoxide (ZPO, Assay 70% in propanol, Sigma-Aldrich) was mixed with the chelating agent methacrylic acid (MAA, C4H6O2, Assay 98%, Aldrich); this approach is necessary to stabilize the zirconium (Zr) precursor. Both blends were mixed for 40 min before being combined together. 1 wt. % of 4,4-bis(diethylamino)benzophenone (Michler’s ethyl ketone) photoinitiator (Sigma Aldrich) was added and mixed for 6 hours. The resulting liquid sol-gel composite photo-active resin was filtered through a 5 μm syringe filter to remove any inclusions that could affect laser polymerization. Substrates for scaffold fabrication were prepared by drop casting of 1 mL of liquid material onto 22×32 mm glass substrates, followed by slow evaporation of the organic solvent over 24 h at room temperature and subsequent baking at 100°C for 2 h. Approximately 300 μm thick photopolymer layers on a glass substrate were produced using this method.

### 2PP fabrication of scaffolds

2PP fabrication of scaffolds was performed using Ti:sapphire femtosecond laser system (Chameleon, Coherent, Germany), which delivers 150 fs pulses at an 80 MHz repetition rate. The experimental setup is similar to one that was previous described (Ovsianikov et al., 2008). An acousto-optical modulator was used to trigger exposure, while a quarter-wave plate mounted on a rotational stage and a polarizing beam splitter were used to modulate the average laser power delivered to the sample. The beam was cleaned using a pinhole spatial filter after these two components. A galvano-scanner (hurryScan10, Scanlab, Germany) was used to finely control the x-y laser position within a small field-of-view. Finally, the modulated laser beam was passed through a 20x microscope objective (objective information Zeiss Epiplan ×20, NA: 0.4) and focused into the photosensitive resin. The long-range x-y adjustment (e.g., between components of the scaffold) and the sample-objective separation distance were controlled with piezo-electric translation stages (M-686K004) and a DC-motor (M-126.DG1) respectively (Physik Instrumente (PI) GmbH & Co, Karlsruhe, Germany). Scaffolds were fabricated using a custom-written computer code to produce the desired geometries from user-defined fabrication parameters.

**Fig 1:**
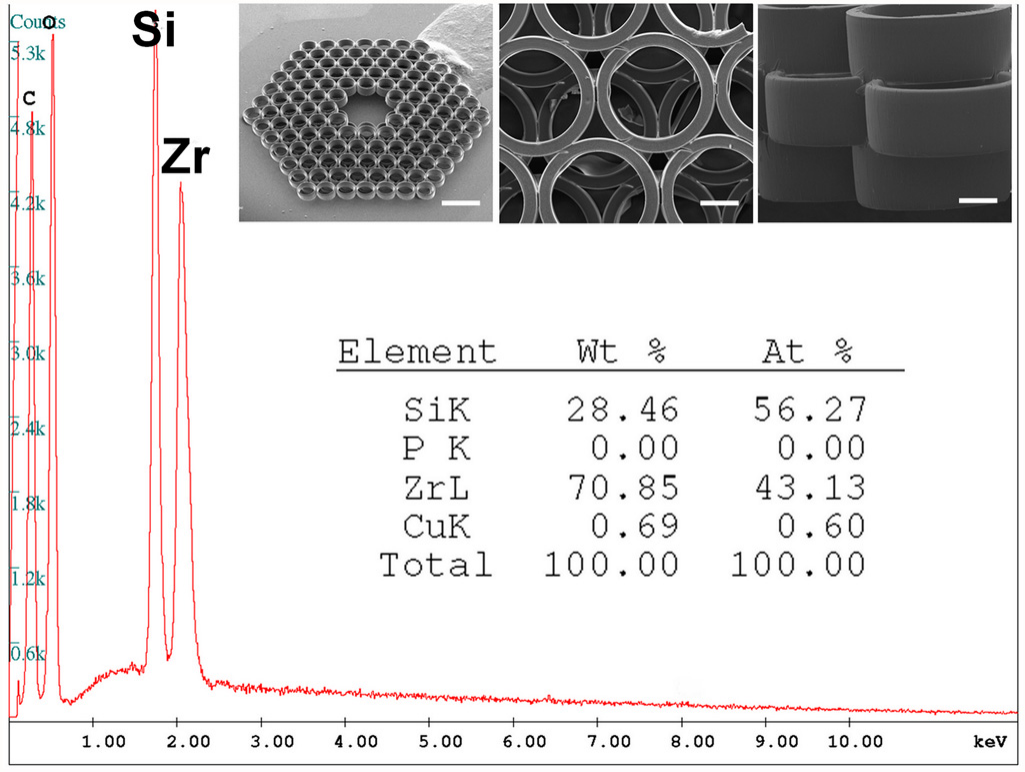
EDX spectrum and SEM demonstration of 2PP fabricated Zr-Si inorganic-organic hybrid scaffold with a pore size of 250 μm. Scale bars: 500 μm, 100 μm and 50 μm for the left, middle and right SEM images, respectively.

Scaffolds were composed of three layers of hollow cylinder arrays (Fig. 1). The outer radius of each cylinder in a layer was defined by the scaffold pore size plus 30 μm wall thickness (e.g., the diameter of each cylinder for a 200 μm pore scaffold would be 260 μm). The cylinders were located in a hexagonal arrangement within each layer. The next scaffold layer was produced by translating the stage with the sample translated 100 μm downwards and the center position translated by the half of the cylinder diameter (x and y shift), before repeating the previously described procedure. The third layer was similarly produced by again translating the sample downwards but shifting the layer center back to the x,y origin. A single scaffold consisting of three layers was produced within 50 min, after that the stage was automatically translated to the position where the next scaffold should be fabricated. 30 scaffolds were produced per 22×32 mm material substrate in a rectangular array. After 2PP fabrication, the samples were developed in 1-propanol to remove the unpolymerized material.

The scaffolds consisted of overall 360, 450 and 702 hollow cylinders for 250, 200 and 150 μm inner cylinder diameters, respectively. The wall thickness of hollow cylinders was kept constant at 30 μm for every pore size. 150 μm was chosen as a minimum pore size for the scaffolds composed of three layers of hollow, shifted to each other, cylinder arrays with constant wall thickness. Due to the offset of cylinder layers, the pores of the scaffold are hierarchically reduced from the top and bottom layers to the scaffold interior. Therefore, further reduction of the pore size would result in the disappearance of porosity in the scaffold inner. Scaffold porosities were calculated as: Porosity % = (V _void_ / V _total_) × 100, where V _total_ is the total volume of the solid hexagonal scaffold figure; V _void_ is the volume of cylindrical holes. Total porosities for 250, 200 and 150 μm scaffolds were 65, 60 and 54%, respectively.

### Mechanical tests of scaffolds

Young’s modulus and hardness evaluation of scaffolds were performed using a “INTEGRA-Terma” system for instrumented nanoindentation with accompanying NanoScan software (JSC “NT-MDT”, Russia). The measuring system contains a nanosclerometrical head with a displacement transducer; it is equipped with a resonance sensor and a triangular diamond sensing device, the Berkovich indenter. The flexural rigidity of the Berkovich indenter is 1 × 103 N/m. Fused quartz with known for Young’s modulus and stiffness values was used as a reference. Measurement ranges for hardness evaluation were 0.1–80 GPa and for Young’s modulus 0.1–3000 GPa. 200 indents were applied for each sample and absolute values of Young’s modulus and hardness were calculated. The calculation was made according to an established approach [19]. The measurements of hardness and Young’s modulus were performed on three groups of 2PP-fabricated scaffolds: after development, after aging in water for one week, and after aging in ethanol for one week. All scaffolds were dried in air prior to the measurements.

### Cell culture

Chemicals and antibodies were purchased from Sigma-Aldrich (Deisenhofen, Germany) unless otherwise noted. Human bone marrow stem cells (hBMSCs) and cell culture media were purchased from Lonza (Basel, Switzerland) and cultured according to supplier instructions in MSC growth medium supplemented with 10% fetal bovine serum (FBS) and 100 units/mL gentamycin. Human adipose-derived stem cells (hASCs) were isolated and cultured as previously described [20]. Briefly, adipose tissue was washed with phosphate-buffered saline (PBS) and then subjected to collagenase digestion. After several filtration, washing, and centrifugation steps, the remaining cells were transferred to cell culture plates in expansion medium consisting of Endothelial-Growth-Medium-2 (EGM-2), and cultured at 37°C in a humidified atmosphere containing 95% air and 5% CO2 for 2 days [21]. Non–adherent cells were removed by medium exchange. Plastic adherent hASCs were maintained in the expansion medium with medium changes produced twice a week. Sub-culturing was performed before the hASCs reached a confluence. hBMSCs and hASCs from the passages 6–7 were used for the scaffold seeding experiments.

### Cell seeding of scaffolds and culture

Before cell seeding, the 2PP produced scaffolds were sterilized by UV light for 30 min. The cell seeding of scaffolds was performed in 96 cell culture well plates. To have the cells preferentially attach to the scaffolds versus the well plate walls, the wells were coated with poly-2-hydroxyethyl methacrylate (polyHEMA). A layer of this soft biocompatible polymer imparts a nonadhesive character to the surface; as such, the seeded cells are prompt to adhere to the scaffolds [22]. hBMSCs and hASCs were harvested from the cell culture plates with 0.05% trypsin/0.02% EDTA. The cells were pelleted by centrifugation at 450 g for 10 min and were resuspended in DMEM (Dulbecco’s modified Eagle’s medium) supplemented with 10% fetal bovine serum (FBS, Biochrom AG, Berlin, Germany), 100 U/mL penicillin, and 100 μg/mL streptomycin. Each scaffold was seeded with 1x10^5^ cells of either hASCs or hBMSCs in 100 μL of cell culture medium.

For each cell type and each scaffold pore size, two experimental groups of scaffolds were maintained: (1) cell seeded scaffolds cultured in osteogenic induction medium consisting of DMEM supplemented with 10% FBS, 1 μM dexamethasone, 50 μM ascorbate-2-phosphate, and 10 mM β-glycerophosphate and (2) a control group of cell-seeded scaffolds cultured in DMEM supplemented with 10% FBS and 1% penicillin–streptomycin only. This experiment was performed for 21 days; differentiation and control media were changed every second day.

### Cell proliferation and viability assays

The potential cytotoxicity of the Zr-Si scaffold material was assessed by measuring lactate dehydrogenase (LDH) leakage into the culture medium caused by cell membrane damage [23]. The amount of LDH released is proportional to the number of damaged or lysed cells. The cells were seeded onto the scaffolds at a density of 1 x 10^5^ in 200 μL of culture medium and incubated for 24 h. The culture medium was then removed and the release of LDH into the supernatant was determined by the LDH activity assay according to the online protocol provided by the manufacturer (OPS Diagnostics, Lebanon, NJ, USA). The absorbance was analyzed using a Tecan Infinite M200Pro microplate reader with Tecan i-controlTM software (Tecan, Crailsheim, Germany) at 492 nm wavelength. Treatment of cells with 1% Triton-X100 served as a 100% positive control of cell damage. The identical number of cells were seeded on tissue culture plastic (TCP) and used as a negative control.

The proliferation of hASCs and hBMSCs seeded on scaffolds was monitored via modified LDH method over 21 days. LDH can be released only from viable cells. Comparison of the measured LDH signal from lysed cells with the control LDH data from known numbers of lysed cell (standard series) enables exact estimation of the cell number on scaffolds at different time points [24]. Proliferation of hASCs and hBMSCs cultured within 3-D Zr-Si scaffolds were characterized using LDH release quantification from lysates obtained after 3, 7, and 21 days of cultivation. At these days, scaffolds were washed three times with PBS. Cell lysis was achieved with 1% Triton X-100/PBS. Ultrasonication of the scaffolds was applied to lyse the cells. After centrifugation at 12000 g for 10 min, the supernatants were removed and used for ALP quantification. The cell number within the scaffolds was determined through the total activity of LDH in the cell lysates. The LDH activity was correlated with the cell number from a cell standard curve, which was prepared in parallel under the same conditions [24–26].

### ALP activity and Calcium assays

ALP activity was measured with the Alkaline Phosphatase-liquicolor assay (HUMAN Diagnostics, Wiesbaden, Germany) according to the manufacturer’s instructions; absorbance of samples was measured at 405 nm wavelength. The ALP value of the samples was quantified by comparison with a standard curve generated from p-nitrophenol standard (10 mmol/L) dilutions. Enzyme activity was expressed as nmol of p-nitrophenol/min/cell. Calcium concentrations were assessed by the colorimetric assay CALCIUM-liquicolor (HUMAN Diagnostics), which was used according to the manufacturer’s instructions, and absorbance of samples was analyzed at 570 nm wavelength. The calcium in the pellets remaining from LDH and ALP assays was extracted in 0.5 N HCl at 4°C for 48 h. The total calcium content was calculated from a standard curve prepared in parallel and was expressed as μg Ca per cell. Each experiment was performed in triplicate.

### Immunohistochemical and histochemical staining

Immunohistochemical staining of the cell seeded Zr-Si scaffolds was performed by a two-step indirect method. Scaffolds were fixed in 4% paraformaldehyde for 30 min. To inhibit unspecific antibody binding, a 2% bovine serum albumin/PBS solution was applied at 37°C for 2 h. After blocking, scaffolds were incubated with primary antibodies diluted in 0.3% Triton X-100/PBS against osteocalcin 1:100 (mouse monoclonal IgG2a, clone C8, Santa Cruz, Heidelberg, Germany) overnight at 4°C. After several washing steps, scaffolds were incubated with horseradish peroxidase-conjugated Goat anti-mouse IgG (H+L) secondary antibody at a 1:100 dilution (Dianova, Hamburg, Germany) for 1 h. After several washing steps, immunostaining with peroxidase was developed by incubation with 3-amino-9-ethyl-carbazole substrate in sodium acetate buffer (0.1 mol/L, pH 5.2) containing hydrogen peroxide. Matched isotype controls IgG2a (clone HOPC-1, Biozol Diagnostica, Eching, Germany) were used to confirm, that the primary antibody binding was specific and not a result of non specific receptor binding or other protein interactions. Histochemical cell staining was applied to visualize ALP activity in the cells via histochemical detection with naphthol AS-MX phosphate as a substrate and Fast Red TR salt as a coupler. Calcium deposits were detected by staining with 2% Alizarin Red S (pH 4.2). Cell nuclei were stained with Hoechst 33342. Scaffolds with cells were analyzed by microscopy using an AxioImager A1.m microscope (Carl Zeiss, Oberkochen, Germany) equipped with an AxioCam ICc1 camera and AxioVision Rel. 4.8 software.

### Scanning electron microscopy

Scanning electron microscopy (SEM) of scaffolds populated with hASCs and hBMSCs was performed at days 2 and 21. For SEM analysis of non-seeded scaffolds, developed and dry structures were sputter coated with 100 nm layer of gold prior to imaging. The cell-seeded scaffolds were washed twice with PBS and fixed with 2.5% glutaraldehyde for 30 min followed by 2% osmiumtetroxide treatment for 30 min. After further washing steps, the scaffolds were dehydrated in a series of ethanol solutions with increasing concentrations (30% to 100%) and finally dried with hexamethyldisilazane (HDMS). For SEM analysis, the cell-seeded scaffolds were sputter coated with a 5 nm gold layer. A silver/carbon sputter coating was applied to the samples that were examined with energy-dispersive X-ray spectrometry (EDX). The scaffolds were examined with a Quanta 400F scanning electron microscope (FEI Company, Oregon, USA), which was equipped with EDX spectrometry (EDAX EDS System and Genesis Software, EDAX Inc., New York, USA), for analysis of cell morphology and for detection of calcium and phosphorus as well as the relative distribution of these elements within the scaffolds.

### Ethics Statement

The Ethics Committee of Hannover Medical School did not specifically approve this study. According the requirements of Ethics Committee of Hannover Medical School (https://www.mh-hannover.de/index.php?id=16578&L=1), no permit is required on studies involving patient endogenous materials, as long as no conclusion to the corresponding patient can be made (anonymized data). Human adipose-derived stem cells (hASCs) were prepared from de-identified human adipose tissue, which was collected from patients undergoing elective plastic surgery after obtaining patients’ written informed consent for their tissue to be used for research purposes.

### Statistical analysis

All data are presented as mean of two independent series of experiments ± standard error of mean (SEM). One hundred scaffolds of each of the three pore sizes (150, 200, 250 μm) were used for cell seeding. 50 scaffolds of each pore size were seeded with hBMSCs and another 50 scaffolds were seeded with hASCs. In both groups, 25 scaffolds were used for osteogenic culture and 25 scaffolds were used for control culture. Statistical significance was determined using analysis of variance followed by a Student’s t-test (p<0.01, 0.05, 0.5).

## Results

### Characterization of Zr-Si scaffolds

The scaffold structure shown in Fig. 1 is characterized by interpenetrating pores with different sizes. The hierarchic decrease of the scaffold pore sizes, starting from the top, is ensured by the offset of each cylinder layer. The top layer of open cylinders may serve to better roping the cells by seeding and their further infiltration into the scaffold interior. The smaller triangular pores in the interstitial space (i.e., between the major circular pores) may serve as routes for fluid transport. EDX analysis of the scaffold composition revealed the presence of carbon, oxygen, copper, silicon, and zirconium with the major content of Si and Zr.

### Viability and proliferation of cells seeded on Zr-Si scaffolds

All groups of scaffolds were seeded with 1x10^5^ hASCs and hBMSCs in polyHEMA-treated 96 well plates. In comparison to conventional scaffold cell seeding procedures, in which a large amount of seeded cells do not reach the scaffold and are cultured on the bottom of TCP, this special cell repellent treatment allowed the scaffold to be the only adherent surface for the seeded cells [22].

**Fig 2:**
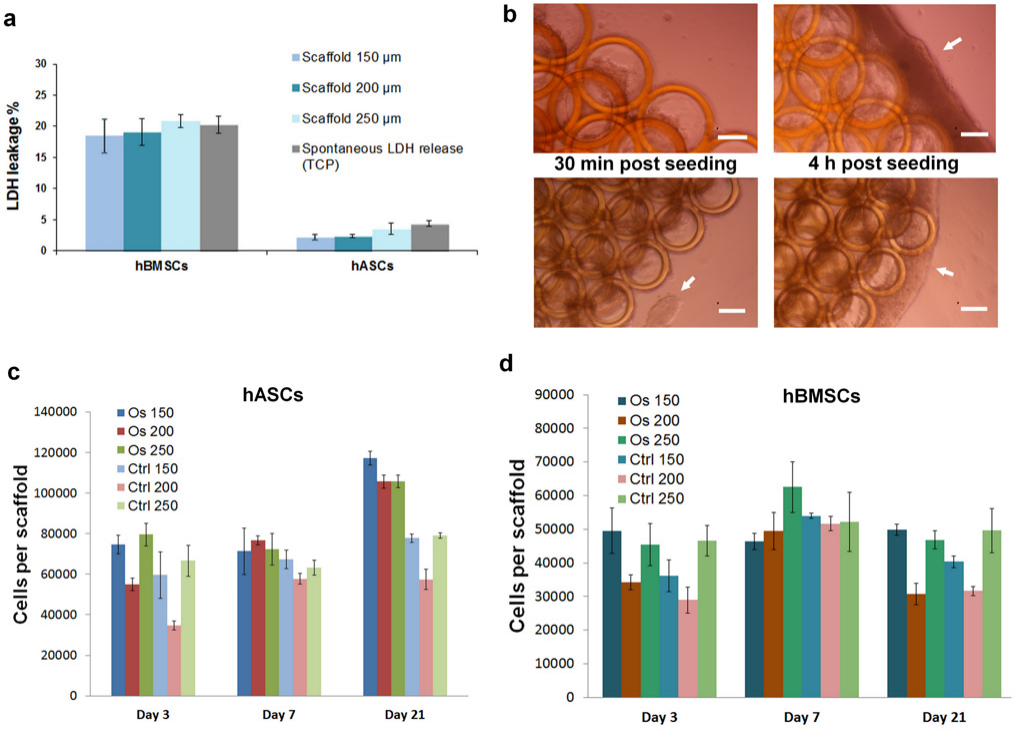
Scaffolds cytotoxicity, seeding efficiency and cell proliferation: a) analysis of scaffold cytotoxicity via LDH assay 24 hours post seeding; b) qualitative demonstration of scaffold population with hASCs 30 min and 4 hours after seeding (scale bars = 100 μm); analysis of proliferation of (c) hASCs and (d) hBMSCs.

For the evaluation of scaffold cytotoxicity, the LDH assay was performed 24 hours after the scaffold seeding. In comparison to hASCs, hBMSCs damage was noted; approximately 20% of initially seeded cells did not survive the seeding procedure (Fig. 2 a). This result cannot be considered as an indicator of scaffold cytotoxicity since spontaneous LDH release from hBMSCs seeded simultaneously on tissue culture plastic (TCP) was as high as LDH release from the hBMSCs seeded on the Zr-Si scaffolds. In comparison to hASCs cultured on TCP, the hASCs showed minimal LDH release when cultured on Zr-Si scaffolds 24 hours after seeding. Right after seeding, the cells showed rapid adherence to the scaffold surfaces, with the majority of cells adhering to the scaffold within 30 minutes; the remaining cells formed spheroids around the scaffold, indicating poor attachment to the well surface (Fig. 2 b, 30 min post seeding, arrow indicates the formation of cell spheroids around the scaffold). After four hours, the cells fully infiltrated the pores of the scaffold and the spheroids began to attach to the external surfaces of the scaffold (Fig. 2 b, arrows). The differences in the pore size did not affect the hASC and hBMSC seeding efficiency; due to the structural characteristics of 2PP scaffolds (e.g., with large interconnected cylindrical pores) hASCs and hBMSCs were well distributed within the entire scaffold.

In comparison to control culture conditions, hASCs cultured on scaffolds in osteogenic media exhibited higher proliferation rates and achieved the highest cell number at day 21 (Fig. 2 c). In contrast, hBMSCs achieved the maximum cell density at day 7, with a general decay in the cell number at day 21. The lowest cell number was measured on the scaffolds with 200 μm pore size for hBMSCs in both experimental conditions (i.e., in osteogenic and in control culture) at days 3 and 21 (Fig. 2 d). Overall, only approximately 50% of the initially seeded hBMSCs could be detected on scaffolds 21 days after seeding.

### Osteogenic differentiation of hASCs and hBMSCs cultured on Zr-Si scaffolds

**Fig 3:**
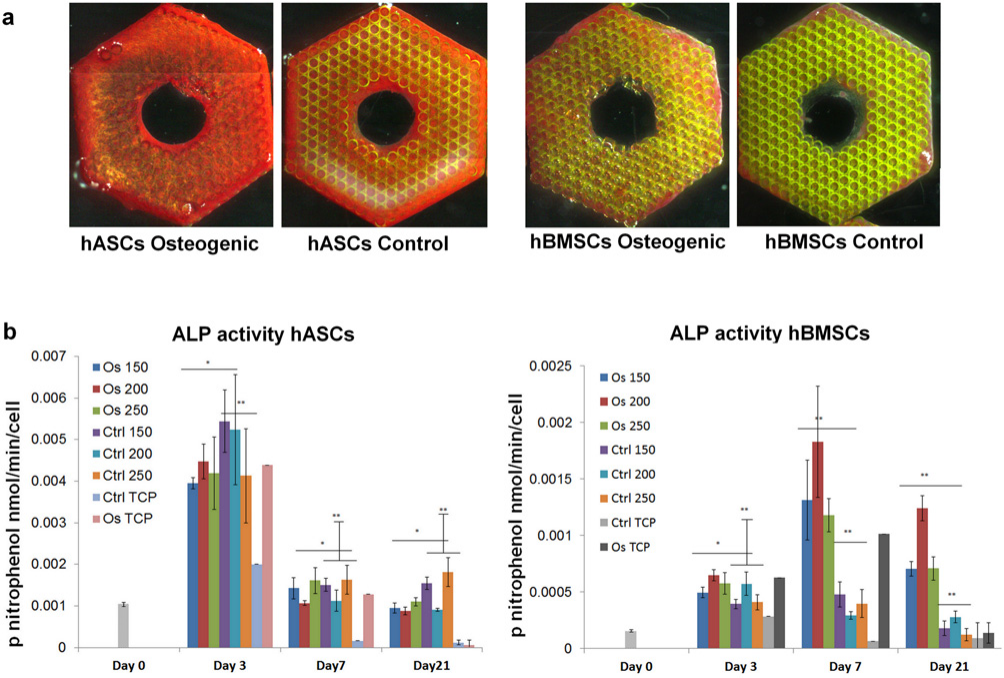
ALP activity of hASCs and hBMSCs cultured on scaffolds: a) qualitative demonstration of histochemical ALP staining of scaffolds at day 7; b) quantified alkaline phosphatase activity of hASCs and hBMSCs cultured on Zr-Si scaffolds. Student’s-t-test: * indicate significant difference of p<0.5; ** indicate significant difference of p<0.01.

ALP activity of hASCs and hBMSCs was monitored over all periods of cell culture on Zr-Si scaffolds. Fig. 3 (a) qualitatively demonstrates ALP staining of stem cells on scaffolds at day 7. It is obvious that ALP staining on the scaffolds seeded with hASCs, in both osteogenic and control media, is stronger than ALP staining on the scaffolds seeded with hBMSCs. Interestingly, the quantitative analysis of the ALP activity indicated that hASCs cultured on scaffolds in the osteogenic medium demonstrated weaker ALP activity than those cultured in the control growth medium. The peak of hASCs ALP activity was reached at day 3; after that, its level steadily decayed (Fig. 3 left). In contrast, hBMSCs cultured on scaffolds in the osteogenic medium revealed significantly stronger ALP activity than the cells in the control medium, and reached the peak of activity at day 7 (Fig. 3 right).

**Fig 4:**
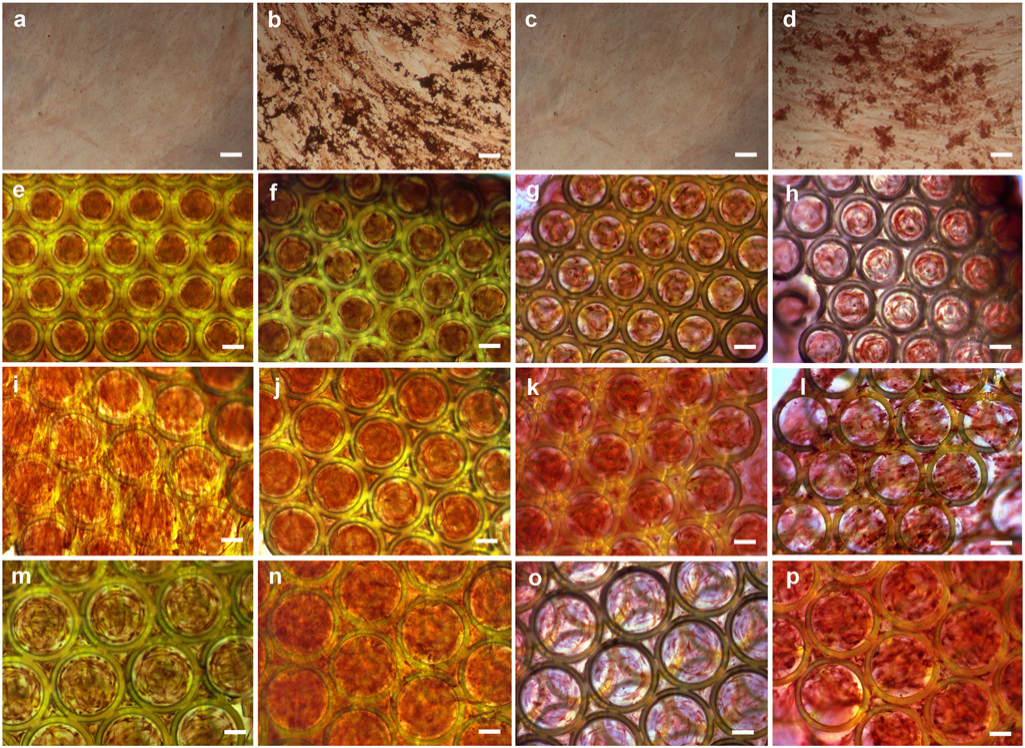
Light microscopy images demonstrating staining on bone matrix specific marker protein osteocalcin. hASCs (a) and hBMSCs (c) cultured on TCP in control media; hASCs (b) and hBMSCs (d) cutured on TCP in osteogenic media; hASCs cultured in scaffolds with 150, 200 and 250 μm pore sizes in control (e,i,m) and in osteogenic media (f,j,n); hBMSCs cultured in scaffolds with 150, 200 and 250 μm pore sizes in control (g,k,o) and in osteogenic media (h,l,p). Scale bars = 100 μm.

In addition to measuring the ALP activity, the expression of osteocalcin protein was used to confirm the osteogenic differentiation of hASCs and hBMSCs cultured on 3-D Zr-Si scaffolds. Since osteocalcin expression is preceded by ALP activity, immunohistochemical analysis was performed at day 21. The osteogenic differentiation of hASCs and hBMSCs has been confirmed by the expression of osteocalcin (Fig. 4). Highly specific immunohistochemical staining demonstrated great amounts of the bone matrix protein osteocalcin on the 3-D Zr-Si scaffolds cultured in both osteogenic and control conditions (S1 Text demonstrates the lack of non-specific antibody binding (mouse IgG2a isotype control)). It should be explicitly noticed, that under control culture conditions and without osteogenic stimulation, osteocalcin expression by cells on 3-D scaffolds was stronger than that by cells cultured on 2-D TCP (Fig. 4 (a,c) vs. (e,i,m) and (g,k,o)):). Overall, the osteocalcin expression by differentiated hASCs was higher than the osteocalcin expression by hBMSCs.

**Fig 5:**
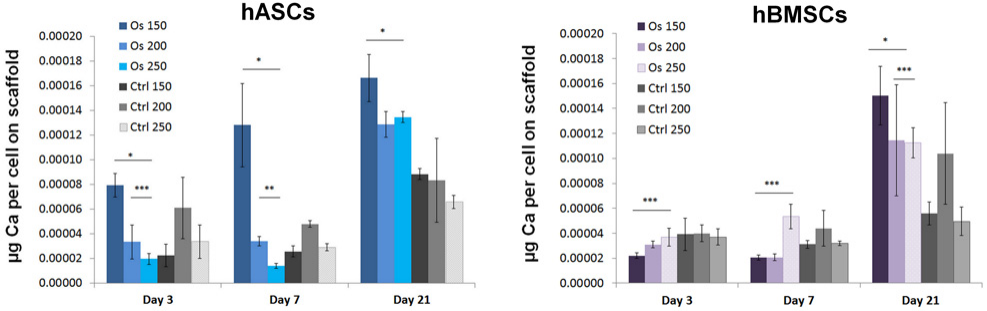
Quantification of calcium deposited by hASCs and hBMSCs in Zr-Si scaffolds during 21 days in culture. Student’s- t-test: * indicate significant difference of p<0.01; ** indicate significant difference of p<0.05, *** indicate significant difference of p <0.5.

Biochemically measured calcium levels are given in Fig. 5. Compared to the scaffolds cultured in the control growth medium, both hASC and hBMSC-seeded scaffolds cultured in the osteogenic medium supported enhanced calcium deposition. The following calcium values were detected at day 21 in the osteogenic culture: (1) 0.00017, 0.00012, and 0.00013 μg Ca produced by cell on scaffolds with 150, 200, and 250 μm pore sizes populated with hASCs, and (2) 0.00015, 0.00011, and 0.00011 μg Ca per cell for scaffolds with 150, 200 and 250 μm pore sizes populated with hBMSCs, respectively (*p* < 0.05). The highest calcium content was observed for both cell types on the scaffolds with 150 μm pore size cultured in the osteogenic medium.

### SEM and EDX mapping analysis calcium phosphate deposition

**Fig 6:**
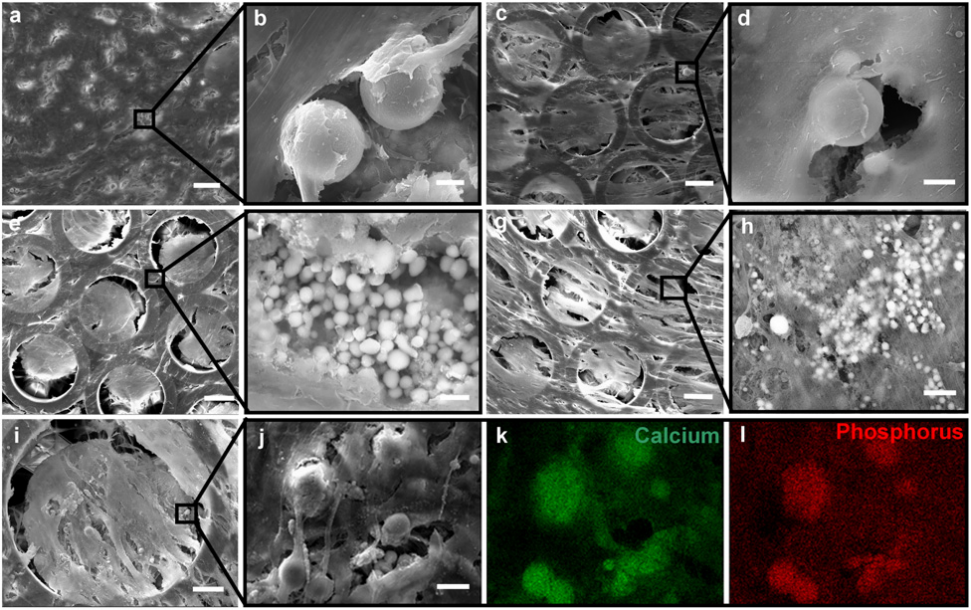
Cells on scaffolds after 21 days in osteogenic and control cultures: (a and c) hASCs and hBMSCs from osteogenic culture; (e and g) hASCs and hBMSCs from control culture. High magnification images showing calcium phosphate deposits of hASCs and hBMSCs cultured on Zr-Si scaffolds in osteogenic (b and d) and control (f and h) conditions; (i-l) EDX mapping confirming the presence of calcium and phosphor in the accumulations. Scale bars: (a, c, e, g) 60 μm, (b, d, f, h) 5 μm, (i) 50 μm and (j-l) 10 μm.

At day 21, both hASCs and hBMSCs cultured on scaffolds were well distributed forming a cell monolayer on top of the constructs (Fig. 6 a, c, e, g). Cells were attached to the scaffold lateral surfaces, and filled the pore spaces. The cellular matrix density appeared to be greater for both cell types in the scaffolds with 150 μm pore size. The cells cultured on scaffolds with larger pore sizes formed a less dense monolayer with fewer cell-to-cell contacts. Cellular deposition of calcium phosphate pellets was observed for both cell types on Zr-Si scaffolds cultured in osteogenic and control conditions. Fig. 6 (b) and (d) shows mineral deposits produced by hASCs and hBMSCs cultured on scaffolds in osteogenic media: 5 to 30 μm small spherical calcium phosphate pellets were shown to accumulate in the pore cavities and distribute throughout the scaffold. Calcium phosphate depositions were also observed for hASCs and hBMSCs on scaffolds cultured in control conditions (Fig. 6 f, h). These mineral deposits were significantly smaller, which indicates delayed onset of bone mineralization on scaffolds cultured in non-stimulated conditions. For verification of calcium phosphate, EDX mapping was used to examine scaffold calcium phosphate accumulations (Fig. 6 j, k, l). EDX mapping detected the presence of Na, Mg, C, O, Ca and P in the deposited accumulations; the accumulations contained high levels of Ca and P (Fig. 6 k, l green and red images represent allocation of Ca and P within deposited by cells mineral matrix).

### Early calcium phosphate deposition on non aged scaffolds

**Fig 7:**
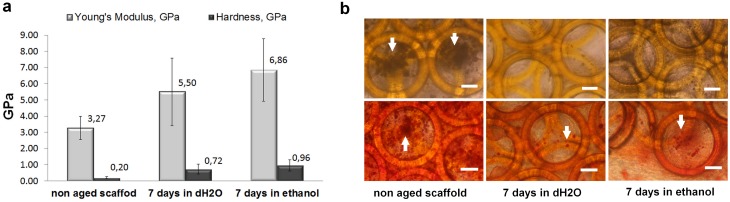
Mechanical properties of the non aged and aged scaffolds (a); Phase contrast (upper row) and Alizarin Red S staining (lower row) of cellular calcium phosphate deposits on non aged and aged Zr-Si scaffolds seeded with hASCs and cultured for 26 days in osteogenic media (b): Arrows indicate calcium phosphate deposits. Scale bars = 50 μm.

Photoinitiator-associated cytotoxicity is the major concern associated with application of photosensitive materials for fabrication of tissue engineering scaffolds. Based on the previous studies that described photoinitiator removal by aging scaffolds in water, we applied an aging method for post-polymerization processing of Zr-Si scaffolds [27]. Hardness and Young’s modulus data were obtained for three groups of scaffolds: non aged, aged in water for 7 days, and aged in ethanol for 7 days. It was found that aging in water and ethanol significantly increases Young’s modulus and hardness values of the Zr-Si scaffolds, making them less elastic (Fig. 7 a). In comparison to the Young’s modulus and hardness values of the non aged scaffolds, aging in ethanol increased the Young’s modulus by 2 fold, and increased the scaffold hardness by almost 5 fold. Aging in water increased the Young’s modulus by 1.7 fold and increased the hardness by 3.6 fold compared to non-treated scaffolds. In a set of preliminary experiments, aged and non aged scaffolds were seeded with hASCs and cultured in osteoinductive media for 26 days. We did not observe material/photoinitiator cytotoxicity. However, earlier deposition of cellular calcium phosphate on non aged scaffolds was noted already at day 4 in comparison to aged scaffolds. After 26 days in osteogenic culture hASCs on non aged scaffolds could build considerable mineralized matrix, whereas hASCs cultured on the aged scaffolds did not deposited significant amount of calcium phosphate pellets (Fig. 7 b). This phenomenon can be associated with the alteration in mechanical properties of the scaffolds, which is attributed to aging of scaffolds in water and ethanol.

## Discussion

In this work, we demonstrated that 2PP can be used to fabricate functional Zr-Si scaffolds for bone tissue engineering. Excellent correspondence between the 2PP-fabricated scaffolds and the computer input parameters was noted. 2PP fabrication enabled the scaffold pore size to be readily modified. Human mesenchymal stem cells from two different tissues, adipose tissue and bone marrow, were seeded and osteogenically differentiated on scaffolds with 150, 200 and 250 μm pore sizes.

The developmental sequence of bone is described by three phases: proliferation with matrix secretion, matrix maturation, and matrix mineralization [28]. In our work, approximately 20% of hBMSCs did not survive the seeding procedure (as indicated by LDH assay 24 hours after scaffold seeding) (Fig. 2 a). hASCs had better seeding characteristics, with a majority of cells surviving the seeding procedure.

The proliferation of hASCs and hBMSCs seeded on scaffolds was monitored via modified LDH method over 21 days. LDH method of proliferation measurement on 3-D scaffolds is advantageous over other methods such as Alamar Blue, DNA or protein assay. On one hand, the lysis of cells allows quantification of all viable cells on scaffold, whereas Alamar Blue dye can be only reduced by cells on scaffold outer surfaces, not reaching the cells in scaffold interior due to the strong ECM building [29]. On the other hand, in comparison to DNA and protein assay, where the data usually represents signals from viable and dead cells, LDH signal is measured only from viable cells and therefore, dead cells present on scaffold are not co-quantificated [30]. As indicated by the proliferation profiles, half of the hBMSCs were lost during the culture and the initial seeding density was not attained at day 21. In contrast, approximately 20% of hASCs were lost at day 3 (Fig. 2 c, d). Moreover, these cells showed better proliferation on the scaffold and the number of the cells exceeded the 10^5^ in osteogenic culture at day 21.

In the matrix maturation phase, the proliferation rate of osteogenic cells decreases and several genes, including those associated with ALP, are expressed [28]. ALP levels peak as cells progress from a proliferative stage to deposition of a mature extracellular matrix containing calcium phosphate [31]. In our study, the peaks of ALP were observed at day 3 for hASCs and at day 7 for hBMSCs (Fig. 3 b), which correspond to the proliferation profiles of these cells. hASCs did not significantly increase their number between the days 3 and 7; hBMSCs reached their maximum cell number at day 7 (Fig. 2 d). Qualitatively, as compared to hBMSCs, the ALP of hASCs was stained considerably stronger on the scaffolds, which were cultured in both osteogenic and control conditions (Fig. 3 a). It has been reported that, ALP is expressed early in the bone development, and can be soon observed on the cell surface and in matrix vesicles. Following osteogenic developmental program, when other genes (e.g. osteocalcin) are upregulated, ALP expression declines. Thus, ALP activity precedes the expression of specific bone proteins in the osteogenic differentiation process and facilitates deposition of ECM before the onset of mineralization [31, 32]. Further differentiation of hASCs and hBMSCs towards osteogenic lineage cultured within the 3-D Zr-Si scaffolds was determined by immunohistochemical osteocalcin staining at day 21 (Fig. 4). It has been observed that hASCs and hBMSCs cultured on 3-D scaffolds in both osteogenic and control media were osteocalcin positive in comparison to the cells cultured on TCP in similar conditions. This result indicates the importance of 3-D scaffold architecture for spontaneous osteogenic differentiation of mesenchymal stem cells and increased bone specific ECM production.

The highest calcium levels were measured for 150 μm pore scaffolds with both hASCs and hBMSCs (Fig. 5). Indeed, in comparison to cell proliferation profiles on 200 and 250 μm scaffolds, hASCs and hBMSCs cultured on the 150 μm pore scaffolds did not significantly proliferate between the days 3 and 7, suggesting that the initially high cell number used for seeding was sufficient for population of complete 150 μm pore scaffolds. The dense distribution of cells within the 150 μm pore scaffolds resulted in closer cell-cell contacts, which subsequently has enabled rapid matrix secretion and accelerated matrix calcification. It has been reported that the direct cell-cell contacts significantly enhance the osteogenic differentiation of BMSCs and the differentiation extent varies with the cell-cell contacts [33]. Gap junctions are small channels that are formed between the contacted cells. These channels are responsible for transduction of signaling molecules from cell to cell; such cell communications play an important role in stem cell differentiation [33–35]. Stronger matrix mineralization was observed by SEM on scaffolds cultured in osteogenically stimulated conditions (Fig. 6 b, d). However, hASCs and hBMSCs cultured on Zr-Si scaffolds without osteogenic stimulation have also started to deposit calcium phosphate. Taken together, the results of positive osteocalcin staining and onset of bone mineralization in control conditions represent the compelling evidence that mesenchymal stem cells cultured on Zr-Si scaffolds spontaneously differentiate towards osteogenic lineage (Fig. 6 f, h).

Numerous studies have shown that the determination of mesenchymal stem cell fate is dependent on mechanical interaction between the cells and matrix on the molecular interface [36, 37]. On stiff matrices, MSCs express four-fold greater osteogenic response, upregulating osteocalcin and other osteogenic transcriptional factors [38]. Recently published scientific investigations involving the influence of scaffold materials on stem cell differentiation deal with soft matter or hydrogel based scaffolds. Reported stiffness values for osteogenesis induction are in the range of 25–50 kPa for various scaffold materials, including collagen, polyacrylamide gels and silk fibroin [37–39]. In comparison to previously reported soft scaffolds and hydrogels, the Young’s moduli values of the 2PP-fabricated Zr-Si scaffolds measured by the nanoindentation method were six orders of magnitude higher (GPa vs. previously reported kPa) (Fig. 7 a); as such, these materials exhibit Young’s moduli values that are similar to those of native trabecular bone [40]. In this study, increased values of Young’s modulus and hardness of aged in water and ethanol scaffolds can be associated with desorption of not crosslinked organic components from Zr-Si organic inorganic hybrid [41].

Effect of porosity and pore size on osteogenesis *in vivo* and *in vitro* can differ significantly. *In vivo*, bone regeneration in scaffolds requires recruitment and infiltration of cells from the surrounding bone tissue, as well as, ingrowth of blood vessels. Therefore, higher scaffold porosities (larger pore size and low surface to volume ratio) enhance osteogenesis as have been proved by numerous studies [42,43]. *In vitro*, increased osteogenesis (higher ALP values and osteocalcin expression) has been reported to be dependent on lower porosity [1]. Most likely this phenomenon is associated with higher surface to volume ratio of scaffolds and consequently with increased scaffold Young moduli and harness. In our study, scaffolds with 150 μm pore size were composed out of larger amount of cylinders, thus being less porous and more consistent, which subsequently increases scaffold Young’s modulus and stiffness values. This result evidences the importance of scaffold architecture in determining its mechanical properties and accelerated *in vitro* formation of bone tissue. SEM observations at day 21 also revealed differences in cell orientation and cell-monolayer formation on scaffolds with 150, 200 and 250 μm pore sizes. Here again, both hASCs and hBMSCs could build denser cell monolayers on scaffolds with 150 μm pore size and thus formed thicker mineralized matrices (Fig. 6 a). Whereas, in our work, 150 μm pore size of Zr-Si scaffold has been identified as optimal for stem cell based *in vitro* bone TE, the ideal pore size of these scaffolds for *in vivo* bone repair has to be evaluated by their implantation into bones of large mammalian species.

In clinical application, stem cells from adipose tissue have many advantages over mesenchymal stem cells as therapeutic tools. These stem cells are available in larger numbers (4 × 10^7^ cells/100 cm^3^ fat aspirate vs. 1 × 10^5^ cells/30 cm^3^ marrow aspirate) and approximately 60% of the ASC population has osteogenic potential [17, 21, 44]. In this comparative study, the hASCs have been shown to be more suitable for scaffold-based bone tissue engineering in terms of cell survival, proliferation, and promotion of bone matrix formation. Compared to hBMSCs, the ALP activity and calcium deposition profiles for hASCs were at least two times higher. Taking into account the better availability of autologous hASCs, this stem cell type can be considered as a more favorable cell source for autologous bone tissue engineering.

## Conclusions

This study has demonstrated the potential of application of 3-D Zr-Si polymer ceramic scaffolds for autologous bone tissue engineering. Laser-based two-photon polymerization technique allows the fabrication and optimization of 3-D Zr-Si polymer ceramic scaffolds in terms of structure porosity and pore dimensions. It has been demonstrated that human mesenchymal stem cells from bone marrow and adipose tissue respond differently to the extracellular environment, by producing bone-like extracellular matrix in a manner that appears to depend on the pore size and stiffness of the scaffolds. Based on quantitative and qualitative data, it has been demonstrated, that mesenchymal stem cells cultured on Zr-Si scaffolds without osteogenic stimulation also differentiate towards osteogenic lineage. These results highlight the importance of adequate scaffold design and scaffold mechanical properties for stem cell based bone tissue engineering. Altogether, the demonstrated ability of 2PP to produce well defined 3-D Zr-Si scaffold architectures and the possibility of osteogenic differentiation of stem cells in these scaffolds are very promising for future bone tissue engineering applications.

## Supporting Information

S1 Fig IgG2a Isotype control antibody stained Zr-Si scaffold populated with hBMSCs possess slight unspecific purple background color (a) in comparison to strongly and apparently stained osteocalcin of hBMSCs on scaffolds from non-stimulated control group (b). (TIF)

S1 Text Supplementary Information. (DOC)
