# Supplementary figures and images for: Osteogenic Differentiation of Human Mesenchymal Stem Cells in 3-D Zr-Si Organic-Inorganic Scaffolds Produced by Two-Photon Polymerization Technique
Source: PLoS One. 2015 Feb 23;10(2):e0118164. doi: 10.1371/journal.pone.0118164 (PMC4338222; doi:10.1371/journal.pone.0118164)

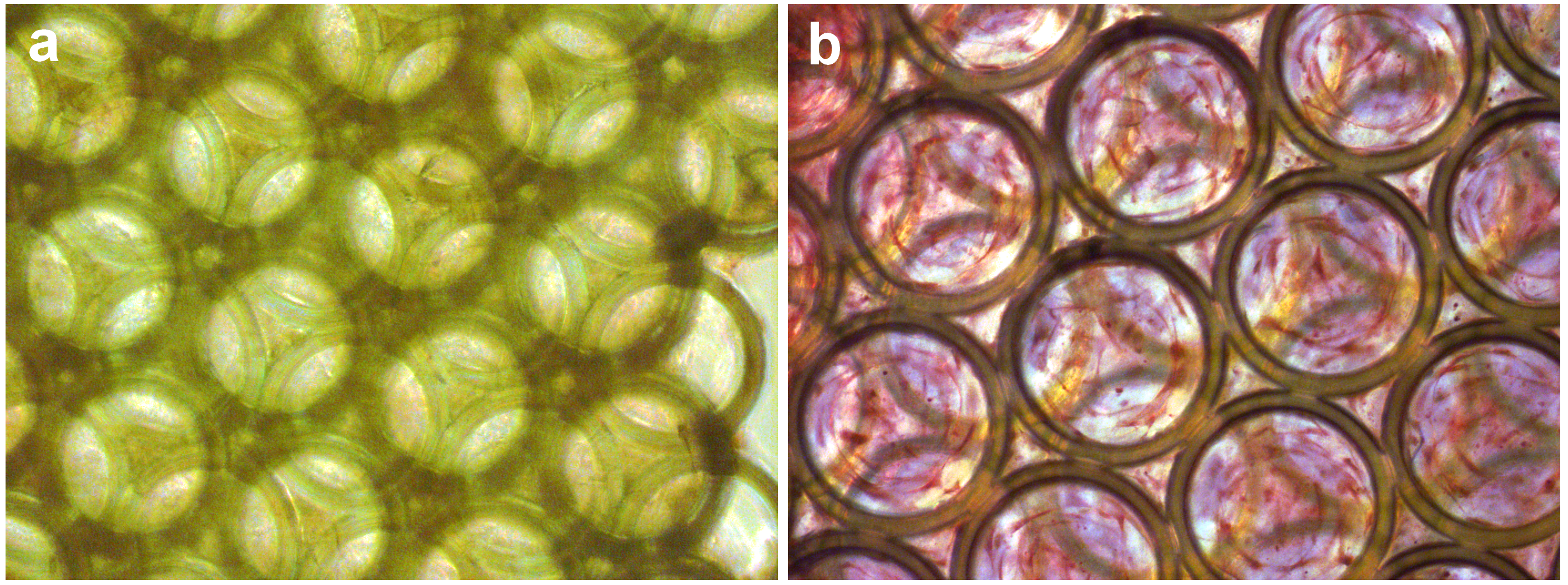

Supplement: S1 Fig — (TIF) [file pone.0118164.s001.tif]
