# Supplementary material for: Osteogenic Differentiation of Human Mesenchymal Stem Cells in 3-D Zr-Si Organic-Inorganic Scaffolds Produced by Two-Photon Polymerization Technique
Source: PLoS One. 2015 Feb 23;10(2):e0118164. doi: 10.1371/journal.pone.0118164 (PMC4338222; doi:10.1371/journal.pone.0118164)
Supplement: S1 Text — (DOC) [file pone.0118164.s002.doc]

**Supplementary information**

Matched isotype controls IgG2a were used to confirm, that the primary antibody binding was specific and not a result of non-specific receptor binding or other protein interactions. An isotype control was included to identify background color. Isotype control antibody was applied at the same concentration as osteocalcin sc-74495 (C-8) antibody.
